# Effects of Acute Partial Sleep Deprivation and High-Intensity Interval Exercise on Postprandial Network Interactions

**DOI:** 10.3389/fnetp.2022.869787

**Published:** 2022-04-13

**Authors:** Zacharias Papadakis, Sergi Garcia-Retortillo, Panagiotis Koutakis

**Affiliations:** ^1^ Human Performance Laboratory, Department of Sport and Exercise Sciences, Barry University, Miami Shores, FL, United States; ^2^ Keck Laboratory for Network Physiology, Department of Physics, Boston University, Boston, MA, United States; ^3^ Clinical Muscle Biology Laboratory, Department of Biology, Baylor University, Waco, TX, United States

**Keywords:** network physiology of exercise, HIIE, postprandial lipemia, heart-rate variability, cardiovascular disease

## Abstract

**Introduction:** High-intensity interval exercise (HIIE) is deemed effective for cardiovascular and autonomic nervous system (ANS) health-related benefits, while ANS disturbance increases the risk for cardiovascular disease (CVD). Postprandial lipemia and acute-partial sleep deprivation (APSD) are considered as CVD risk factors due to their respective changes in ANS. Exercising in the morning hours after APSD and have a high-fat breakfast afterwards may alter the interactions of the cardiovascular, autonomic regulation, and postprandial lipemic systems threatening individuals’ health. This study examined postprandial network interactions between autonomic regulation through heart rate variability (HRV) and lipemia *via* low-density lipoprotein (LDL) cholesterol in response to APSD and HIIE.

**Methods:** Fifteen apparently healthy and habitually good sleepers (age 31 ± 5.2 SD yrs) completed an acute bout of an isocaloric HIIE (in form of 3:2 work-to-rest ratio at 90 and 40% of VO_2_ reserve) after both a reference sleep (RSX) and 3–3.5 h of acute-partial sleep deprivation (SSX) conditions. HRV time and frequency domains and LDL were evaluated in six and seven time points surrounding sleep and exercise, respectively. To identify postprandial network interactions, we constructed one correlation analysis and one physiological network for each experimental condition. To quantify the interactions within the physiological networks, we also computed the number of links (i.e., number of significant correlations).

**Results:** We observed an irruption of negative links (i.e., negative correlations) between HRV and LDL in the SSX physiological network compared to RSX. Discussion: We recognize that a correlation analysis does not constitute a true network analysis due to the absence of analysis of a time series of the original examined physiological variables. Nonetheless, the presence of negative links in SSX reflected the impact of sleep deprivation on the autonomic regulation and lipemia and, thus, revealed the inability of HIIE to remain cardioprotective under APSD. These findings underlie the need to further investigate the effects of APSD and HIIE on the interactions among physiological systems.

## 1 Introduction

A plethora of data suggest the cardioprotective effects of exercise due to its impact either on reducing the associated to cardiovascular disease risk factors (e.g., hypertension, lipidemia, diabetes, and insulin resistance, obesity) or having a direct effect on processes and functions of different physiological systems (e.g., atherosclerotic process and cardiovascular function) (Powers et al., 2002; Phrommintikul et al., 2022). Moreover, exercise presents cardioprotective effects due to autonomic nervous system (ANS) adjustments on heart rate variability (HRV) and baroreflex sensitivity (Grant et al., 2012; Subramanian et al., 2019). High-intensity interval exercise (HIIE), exercise performed in brief, successive intervals consisting of a period of high-intensity (e.g., >80% of peak oxygen consumption) followed by lower-intensity recovery periods, is proposed as alternative time-efficient exercise method with wide health related benefits during both fasting and postprandial states (Gibala, 2007; Wisloff et al., 2007; Tjonna et al., 2008; Tyldum et al., 2009; Gibala et al., 2012; Weston et al., 2014; Bond et al., 2015; Sawyer et al., 2016; Ramírez-Vélez et al., 2018; Tucker et al., 2018). The protective cardiometabolic effect of HIIE is postulated due to greater induced antioxidant status both during and after HIIE (Harris et al., 2008; Di Francescomarino et al., 2009; Fisher-Wellman and Bloomer, 2009; Tyldum et al., 2009; Gabriel et al., 2012). On top of that, HIIE has a significantly greater impact on ANS (Gibala et al., 2012; Bhati and Moiz, 2017), with HIIE work to rest (W:R) ratio of 1:2 to be proposed as highly effective for cardiovascular and autonomic related health benefits (Heydari et al., 2013; Ramírez-Vélez et al., 2016; Ramirez-Velez et al., 2020). It has been shown that ANS disturbance due to exercise, presented as increased sympathetic tone and parasympathetic withdrawal leads to a decreased HRV, which in turn increases the risk for cardiovascular disease (CVD) (Besnier et al., 2017). HIIE affects simultaneously the interactions among physiological systems and organs where the strength of these interactions may represent different physiological states and pathological conditions. Such pathological conditions may be manifested due to failure of the system to perform various coupling and feedback interactions under an integrated physiological system with linear and non-linear characteristics (Bashan et al., 2012; Ivanov and Bartsch, 2014; Bartsch et al., 2015).

Postprandial lipemia is an independent risk factor for CVD (Hyson et al., 2003; Cromwell et al., 2007). Postprandial increased concentration of low-density (LDL) lipoprotein cholesterol induces a heightened inflammatory state in the vascular wall that is highly susceptible to oxidative changes (Littlefield and Grandjean, 2015) promoting vascular endothelial dysfunction (Wallace et al., 2010). In apparently healthy men, LDL cholesterol had inverse relation to HRV (Kupari et al., 1993; Christensen et al., 1999), which is a marker of ANS activation and linked to CVD (Thayer et al., 2010; Chung et al., 2020).

Short sleep duration has been linked to increased risks of morbidity and mortality (Hale et al., 2020; Krittanawong et al., 2020). Acute partial sleep deprivation (APSD), of less than 5 h of sleep, is associated to CVD (Tobaldini et al., 2017; Liew and Aung, 2021). This association to CVD is attributed to changes in autonomic nervous system (ANS) (Miller and Cappuccio, 2013; Tobaldini et al., 2014; Wright et al., 2015; Tobaldini et al., 2017; American Sleep Association, 2018; Seravalle et al., 2018; Liu and Chen, 2019; Oliver et al., 2020). Under APSD such detrimental health effects may represent the breakdown of dynamic network interactions among organs systems and metabolic processes, such as ANS and postprandial lipemia and their failure to ensure a healthy vital status (Bartsch et al., 2015; Ivanov et al., 2016; Balague et al., 2020; Lehnertz et al., 2020). It seems that after sleep deprivation the pronounced HR and HRV reduction reflect the inability of the cardiovascular system to respond and adapt to such a trigger (Zhong et al., 2005; Sauvet et al., 2010). When there is failure in the interaction and coordination between the parasympathetic activity (PA) withdrawal and decreased total HRV (i.e., decreased high frequency, increased low frequency, increased low frequency/high frequency ratio) then sleep deprivation may pose as a risk factor for CVD (Zhong et al., 2005; Tobaldini et al., 2014; Johnston et al., 2020).

It is possible that APSD prior to exercise to mask the intended health benefits of HIIE (Gibala, 2007; Wisloff et al., 2007; Harris et al., 2008; Tjonna et al., 2008; Di Francescomarino et al., 2009; Fisher-Wellman and Bloomer, 2009; Tyldum et al., 2009; Gabriel et al., 2012; Gibala et al., 2012; Heydari et al., 2013; Weston et al., 2014; Bond et al., 2015; Ramírez-Vélez et al., 2016; Sawyer et al., 2016; Ramírez-Vélez et al., 2018; Tucker et al., 2018; Ramirez-Velez et al., 2020), especially for those who are regular good sleepers particularly as it is related to postprandial HRV and cardiometabolic health (Christensen et al., 1999; Gielen and Hambrecht, 2005; Mestek et al., 2006; Mestek et al., 2008; Gielen et al., 2010; Gielen et al., 2011; Chung et al., 2020). Research questions related to the immediate HRV responses after acute HIIE following reference sleep and APSD are still under investigation. Previous work from our lab has examined such research questions (Papadakis et al., 2020; Papadakis et al., 2021a; Papadakis et al., 2021b) under the traditional Exercise Physiology approach, which focuses on a single physiological system by reducing complex multicomponent systems on their respective parts (Machamer et al., 2000; Bechtel and Richardson, 2010; Balague et al., 2020). We reported that the expected HRV disturbance as a response to an acute HIIE was not influenced by APSD (Papadakis et al., 2021a), HIIE after APSD was still cardioprotective for the postprandial endothelial function (Papadakis et al., 2020), and lastly that fasted HIIE and performance were not affected by sleep conditions (Papadakis et al., 2021b).

The human organism though, is comprised by multicomponent physiological systems that operate through non-linear feedback mechanisms at several spatio-temporal scales generating complex dynamics that continuously adapt to various intrinsic and extrinsic stimuli (Bashan et al., 2012; Ivanov and Bartsch, 2014; Bartsch et al., 2015). Accordingly, exercising in the morning hours after APSD may impact negatively interactions among physiological systems (e.g., cardiovascular and ANS) and, therefore, generate a differentiated network of physiological interactions compared to exercising after a full night sleep. It is possible, that many people after exercise, may consume a typical American high-fat convenience breakfast (Bourland and Vogt, 2009; Lang et al., 2014), a behavior that adds another level in the complex interactions between the aforementioned physiological systems. Such behavior, may disrupt the network interactions among the cardiovascular, autonomic regulation, and postprandial lipemic systems ultimately jeopardizing their health.

Therefore, this paper attempts to revisit our previous work (Papadakis et al., 2020; Papadakis et al., 2021a; Papadakis et al., 2021b), through the prism of Network Physiology of Exercise (NPE) (Balague et al., 2020), a new branch of the interdisciplinary field of Network Physiology (Ivanov et al., 2016; Ivanov, 2021; Ivanov et al., 2021). NPE addresses the fundamental question of how physiological systems coordinate and synchronize their dynamics as a network to optimize organism function, and how these network interactions change in response to exercise and training. NPE utilizes novel methods and approaches in Network Theory, Nonlinear Dynamics, Computational and Statistical Physics, and Biomedical Informatics to represent localized integrated organ systems and their interactions across various scales with the respective nodes (examined variables) and edges/links (respective interactions) in their dynamic network (Bartsch et al., 2014; Ivanov and Bartsch, 2014; Bartsch et al., 2015; Ivanov et al., 2016; Ivanov et al., 2017; Balague et al., 2020; Lehnertz et al., 2020; Meyer, 2020; Rizzo et al., 2020). Accordingly, we aimed to examine the postprandial network interactions between autonomic regulation through HRV and lipemia through low-density lipoprotein (LDL) cholesterol in response to APSD and HIIE.

## 2 Materials and Methods

### 2.1 Study Design and Participants

**FIGURE 1:**
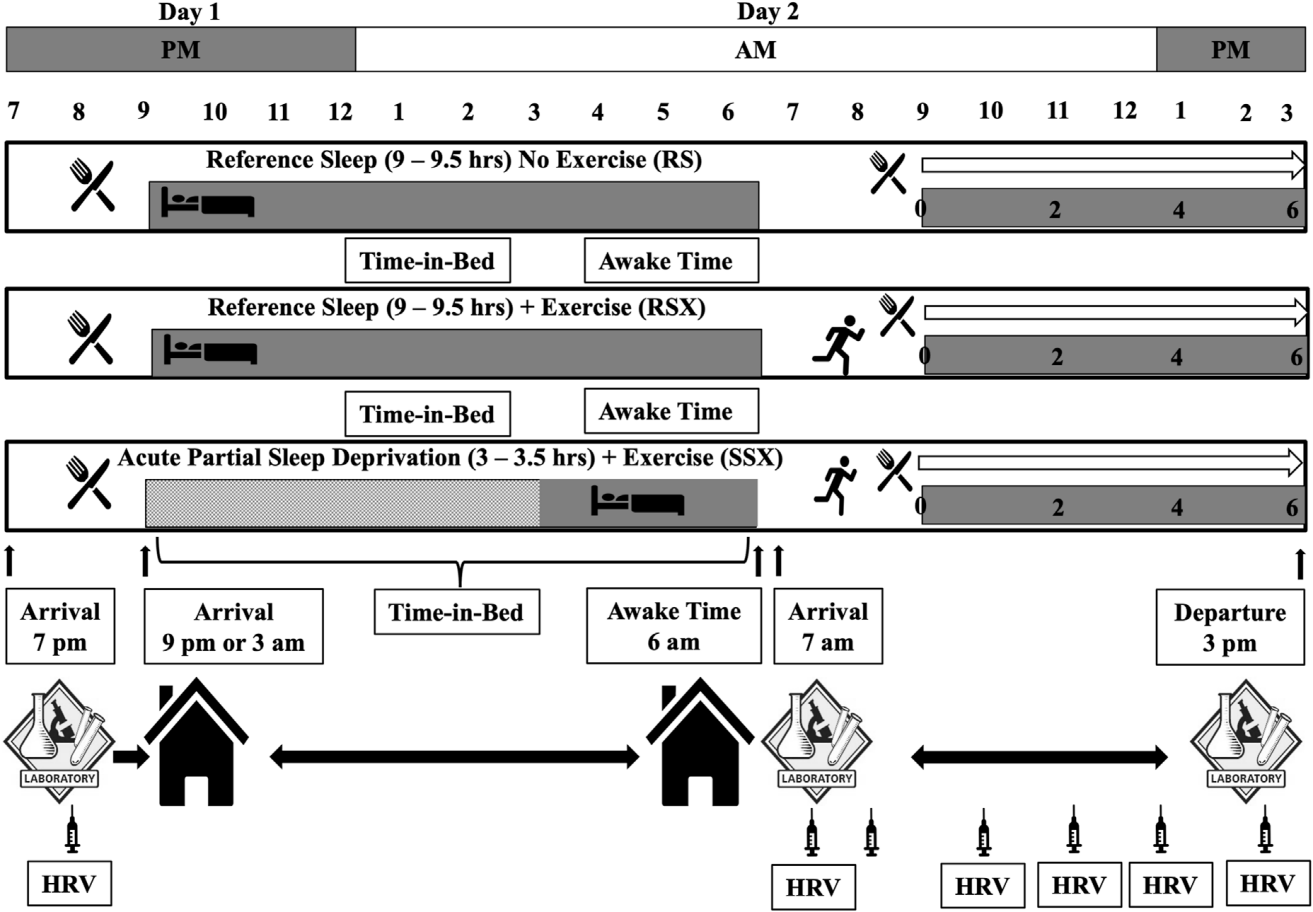
Original Experimental conditions.

As stated, this paper is revisiting data collected as part of a bigger project that involved parameters related to sleep, exercise, and cardiovascular function and outcomes of these investigations presented in detail elsewhere (Papadakis et al., 2020; Papadakis et al., 2021a; Papadakis et al., 2021b). Briefly, a within-subject randomized crossover experimental design with three 3) experimental conditions (i.e., a reference sleep—no exercise “control condition” (RS) in which a standardized test meal was ingested in the morning after 9–9.5 h of time-in-bed in which at least 8 h of sleep was attained; a “reference sleep and high-intensity interval exercise condition” (RSX), similar to RS condition in terms of the meal and the obtained sleep time with the exception of a high-intensity interval exercise with 3:2 intervals at 90 and 40% of VO_2_ reserve that averaged 70% of VO_2_ reserve and expended 500 kcals of energy, and; a “short and disrupted sleep and high-intensity interval exercise—acute partial sleep deprivation condition” (SSX), similar to RSX in terms of the meal and the performed exercise with the exception of the sleep time that was regulated to 3–3.5 h of time-in-bed limited to no more than 3.5 h of sleep) was employed to answer the research questions as depicted earlier (Figure 1). All experimental conditions began after 48 h of controlling activities of daily living, medication use, standardized diet to what the individuals consumed during the first experimental condition, and supplementation of any kind with a minimum 72-h and maximum 2 weeks washout period between each condition. Thirty healthy males (25–55 years) with normal and overweight body mass index (BMI) met the following inclusion criteria: 1) being recreationally physically active, but not engaging in training for long-distance endurance events, 2) non-smokers, 3) not taking any medications known to alter blood pressure, lipidemic and glucose profile, and 4) not taking any medications known to alter sleep. Participants had to be “good” sleepers as indicated by a score of ≤5 on the Pittsburgh Sleep Quality Index (PSQI) (Buysse et al., 1989). Study was approved by the Institutional Review Board and performed in agreement to the Declaration of Helsinki with all participants having read and signed an informed consent form prior to participation.

Experimental conditions completed on two consecutive days and began after 48 h of physical inactivity, no medication use, and the consumption of a diet standardized to what the individual consumed during the first intervention and free from supplementation of any kind. Conditions involved one pre-sleep standard meal consumed in the evening of the first experimental day, six hear rate variability (HRV) recordings, and a morning standard meal surrounding the sleep and exercise interventions. Conditions included: 1) a reference sleep—no exercise “control condition” (RS) in which a standardized test meal was ingested in the morning after 9–9.5 h of time-in-bed in which at least 8 h of sleep was attained) a “reference sleep and high-intensity interval exercise condition” (RSX) in which the test meal was ingested in the morning after reference sleep and after a session of high-intensity interval exercise (3:2 intervals at 90 and 40% of VO_2_ reserve that average 70% of VO_2_ reserve) to expend 500 kcals of energy, and; 3) a “short and disrupted sleep and high-intensity interval exercise condition—acute partial sleep deprivation” (SSX) in which an experimental test meal was ingested in the morning after 3–3.5 h of time-in-bed limited to no more than 3.5 h of sleep and after a session of high-intensity interval exercise to expend 500 kcals of energy. Participants arrived at 7p.m. at the laboratory and stayed around 8:30p.m. During this time blood was collect (indicated by the syringe) and an HRV measurement was taken before the evening meal. We discharged participants from the lab accounting for commuting time and bed-preparation time so at 9p.m. all to be in bed. Participants stayed in their homes until the awake time that it was set at 6:00a.m. During this time only data from Sensewear were collected to verify the sleep duration. Participants had to be at the lab at 7:00a.m. the next day. Between 7 and 9a.m., another blood draw was performed and a HRV measurement was taken around 7:00a.m., followed by the exercise condition and another blood draw immediate post-exercise. Around 8:30–9:00a.m. the morning meal was provided followed by another blood draw and a HRV measurement. Every 2 hours post-exercise a blood draw was performed and an HRV measurement was taken until round 3:00p.m.

### 2.2 Pre-Experimental Conditions

#### 2.2.1 Body Composition and Cardiovascular Fitness

Preliminary measurement of participants’ body composition via dual-energy X-ray absorptiometry (DXA) (Discovery DXA^™^, Hologic^®^, Bedford, MA) was performed. After that, an individualized maximal graded exercise test using a modified ramped treadmill protocol to determine participants’ cardiovascular fitness (VO_2_) via collection of respiratory gases (TrueOne 2400^™^, ParvoMedics^®^, Sandy, UT) was executed. Results of this test were used to calculate the experimental exercise intensities as illustrated in Figure 1 (i.e., 3:2 intervals at 90 and 40% of VO_2_ reserve that average 70% of VO_2_ reserve; VO_2_ reserve was calculated as (VO_2_ max—VO_2_ rest) X % intensity + VO_2_ rest; with VO_2_ rest to be 3.5 ml/kg/min) (American College of Sports Medicine, 2013), and also to familiarize participants with exercise intervals to ascertain their comfort during the experimental conditions.

#### 2.2.2 Sleep and Physical Activity Monitor—Diet Records

On top of using the PSQI scale to identify the “regular good sleepers”, participants’ sleep was monitored by the Sense Wear armband (Sense Wear™, Body Media®, Pittsburgh, PA), which is a validated method to assess both sleep and physical activity parameters (Almeida et al., 2011; Van Wouwe et al., 2011; Sharif and Bahammam, 2013; Soric et al., 2013; Shin et al., 2015). Participants had to wear the monitor on their non-dominant arm for 23 h/day each day, for 1 week prior to experimental conditions and for 2 days leading up to experimental conditions. Information from the monitor was used to characterize participants’ sleep duration, sleep consistency, sedentary time, levels of physical activity and to ensure the homogeneity of study’s sample in terms of sleep and physical activity patterns. Moreover, participants’ diet was controlled as they were asked 2 days prior and during the experimental conditions to maintain their typical dietary habits and consume food that were easily reproducible. Dietary intake and macronutrient composition was analyzed using the ChooseMyPlate® (U.S. Department of Agriculture, Washington, DC). Ensuring a stable diet, sleep, and physical activity patterns-habits was paramount to reduce their respective influence on changes in the dependent variables.

### 2.3 Experimental Conditions

#### 2.3.1 Standard Evening Meal

The standardized consumed evening meal of day 1 (∼805 kcal) was turkey and cheese sandwich on whole grain bread, a medium banana, a 150 g cup of Greek yogurt, and a 24 oz Gatorade® drink. It was the last meal that all participants had from 7p.m. until 9p.m., before they went to bed and until the following morning. Participants remained at the lab until they returned to their residence to go to sleep.

#### 2.3.2 Sleep

Participants completed all the sleep elements of the study at their residence in order to eliminate any disturbances that may occurred if they slept in an unfamiliar laboratory place. Per research design, the RS and RSX conditions allowed for 9.5 h of time-in-bed in the hopes that at least ≥8 h of sleep would have achieved. In the SSX, the research design allowed for 3.5 h of time-in-bed limiting the sleep to ≤3 h. Researchers instructed participants to return to their residence once they left the lab, as all the experimental conditions were calculated to allow enough time for commute and sleep preparation/hygiene routine. No other food was allowed, no watching television neither engaging in computer activities were allowed as participants were preparing for sleep. Participants had also to record both the time that they entered the bed and the time they woke up.

#### 2.3.3 High-Intensity Interval Exercise

High-intensity interval exercise sessions were performed on Trackmaster^®^ TMX 428CP treadmill. Following the research design, sessions began at least 10–12 h after the evening meal and completed 1 h before the test meal. After a 5-min warmup at 2 mph and 0% grade, the HIIE sessions were completed in 3-min running intervals at 90% of VO_2_ reserve separated by 2-min intervals of jogging/walking at 40% of VO_2_ reserve until 500 kcal were expended. The average intensity of all HIIE sessions was equated to 70% of VO_2_ reserve. The applied HIIE protocol of 3:2 min work to rest ratio was a modified one from previous studies (Kaikkonen et al., 2008; Matsuo et al., 2014; Osuka et al., 2017; Ito, 2019).

#### 2.3.4 Standard Morning Meal

A standard commercially available meal was provided to participants 60 min after completing the HIIE sessions of day 2. The meal included a Jimmy Dean^®^ sausage, egg, and cheese biscuit (∼410 kcals; 29 g fat; 26 g carbohydrate; 11.5 g protein); a Jimmy Dean^®^ fully-cooked pork sausage patty (∼270 kcals; 24 g fat; 2 g carbohydrate; 10.5 g protein); a Little Debbie^®^ honey bun (∼483 kcals; 27 g fat; 55 g carbohydrate; 5.5 g protein), and; a cup of whole milk (∼146 kcals; 8 g fat; 11 g carbohydrate; 7.5 g protein). The test meal had a total of 1,309 kcals (88 g fat; 94 g carbohydrate; 35 g protein). For the RS condition of day 2, the meal was provided 60 min after their arrival at the lab and matched their respective HIIE sessions.

#### 2.3.5 Cardiac Autonomic Regulation—Heart Rate Variability

A standardized HRV protocol and methodology for circadian influence on cardiac autonomic assessment was followed as previously described (Malik, 1996; Sammito and Böckelmann, 2016; Riganello et al., 2019; Johnston et al., 2020). Participants after being in supine position for 10 min in a quiet and temperature-controlled environment (∼21–24°C and 40–60% relative humidity), heart rate (R-R intervals) using a Polar belt (FT1^™^, Polar Wearlink^®^ Lake Success, NY) was recorded for 5 min at a sampling rate of 1,000 Hz. We used this heart rate recording to calculate the R-R interval and obtain the related heart rate variability indices as previously described (Achten and Jeukendrup, 2003; Engström et al., 2012; Buchheit, 2014). Cardiac autonomic modulation through HRV was assessed the night before (D1), the morning of the next day (D2), 0, 2, 4, and 6-h post-exercise (PE) (Figure 1). We followed previously published guidelines to reduce anxiety of measurement and control for recording errors (Malik, 1996; Sammito and Böckelmann, 2016; Johnston et al., 2020). CardioMood^®^ smartphone application for iPhone was used to process the recorded data as described (Flatt and Esco, 2015; Markov et al., 2016; Baevsky and Chernikova, 2017; Perrotta et al., 2017). A total of five HRV parameters and time intervals were selected based on the literature (Malik, 1996; Trimmel et al., 2015; Abreu et al., 2019; Johnston et al., 2020). Specifically, 1) the standard deviation of RR interval (SDNN; i.e., marker of the sympathovagal balance influenced by the sympathetic activity) and 2) the root mean square of successive normal RR interval differences (RMSSD; i.e., marker of parasympathetic activity) were examined from the time domain of HRV indices. In addition, 3) the high frequency power (0.15–0.40 Hz) (HF; i.e., marker of parasympathetic activity), 4) total power (0–0.4 Hz) (TP; i.e., marker of the sympathovagal balance influenced by the sympathetic activity), and 5) low frequency power (0.04–0.15 Hz) (LF; i.e., that reflects sympathovagal balance, baroreceptor reflex activity or neither) (Akselrod et al., 1981; McCraty et al., 2001; Rahman et al., 2011; Martelli et al., 2014) were examined from the frequency domain of the HRV indices.

#### 2.3.6 Blood Collection and Biochemical Analysis

Blood samples were drawn using universal procedures (WHO, 2010) as follows: on the evening before rest/sleep (D1); on the following morning just prior to exercise (D2); following exercise immediate post-exercise (IPE) and immediately prior to eating a standard test meal (0h-), and; again at two (2h-), four (4h-), and 6 h post-exercise (6h-PE) after eating the test meal (Figure 1). All assays were performed in duplicate on first thaw of the samples after being stored at -80°C. All blood samples were analyzed for lipemia related variables (e.g., triglycerides, low-density lipoprotein cholesterol, high-density lipoprotein cholesterol, total cholesterol) using commercial ELISA kits (Wako Pure Diagnostics^®^ Richmond, VA). Standard curves for all assays were developed to determine the concentrations in the study samples. All blood variables were corrected for plasma volume shifts known to occur with exercise. All assays for each subject were run on the same day with the same reagent batch to minimize intra- and inter-variability and keep high the internal quality control of our laboratory analysis.

### 2.4 Data Analyses

As noted earlier, the dataset for this manuscript was based on previous work and all the related statistical analyses are described in detail elsewhere (Papadakis et al., 2020; Papadakis et al., 2021a; Papadakis et al., 2021b). We used LDL cholesterol, a marker of cardiovascular risk, due its important clinical significance in cardiovascular disease (Cromwell et al., 2007; Trejo-Gutierrez and Fletcher, 2007), exercise (Durstine et al., 2002), and HRV interactions (Kupari et al., 1993; Christensen et al., 1999; Thayer et al., 2010; Chung et al., 2020; Vijayabaskaran et al., 2022). Since we wanted to investigate the postprandial network interactions between autonomic regulation via HRV and lipemia via LDL under APSD and after HIIE, we constructed one correlation analysis and one physiological network only for the experimental conditions of RSX and SSX. The RSX network was defined as the healthy network. We used two groups of parameters: 1) the five HRV parameters (SDNN, RMSSD, HF, TP, and LF) assessed in six different occasions (D1, D2, 0, 2, 4 and 6-h post-exercise), and 2) LDL obtained in seven different moments (D1, D2, IPE, 0, 2, 4, and 6-post-exercise) (total of 37 parameters).

**FIGURE 2:**
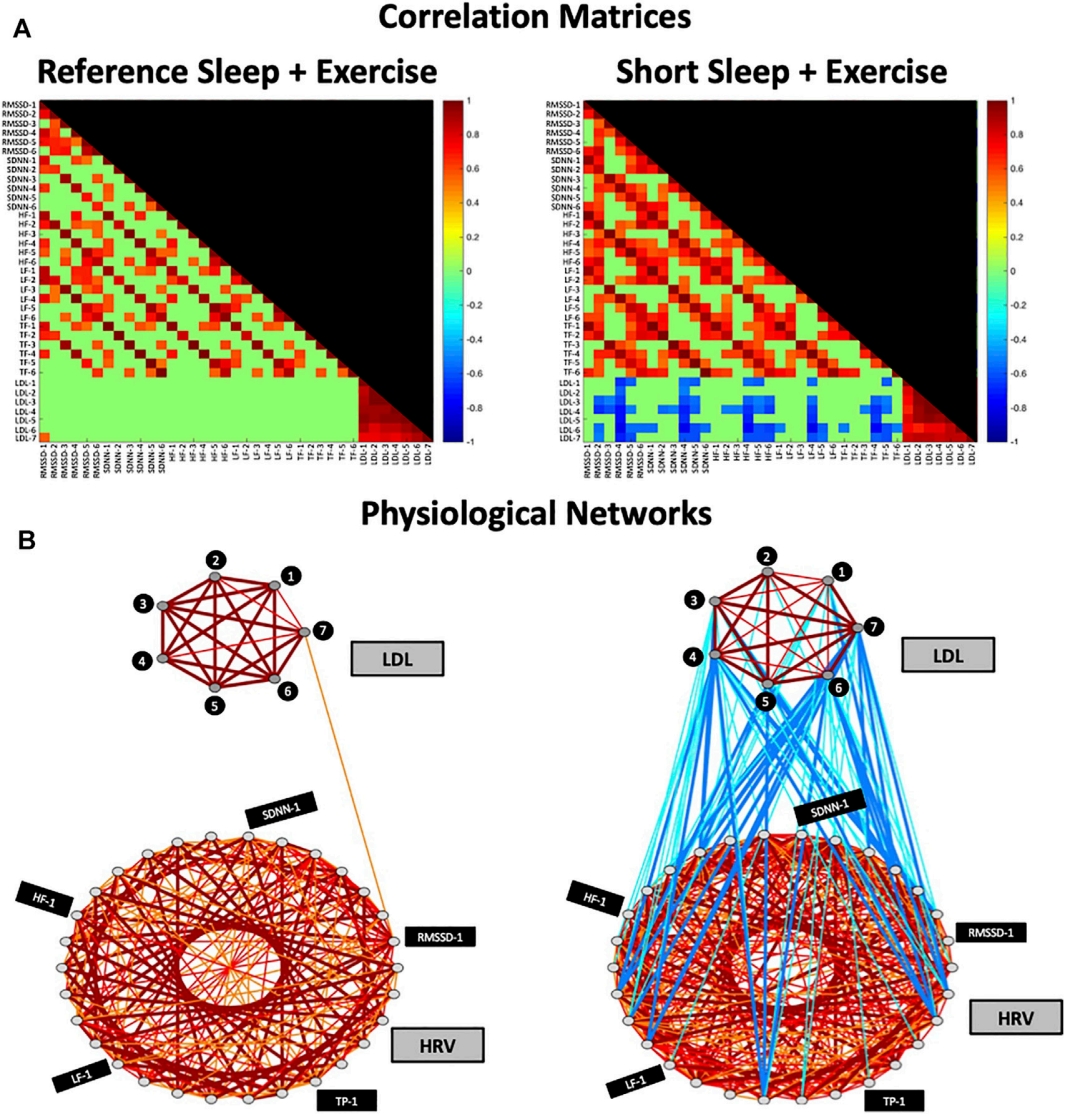
Correlation Matrices and Physiological Networks representing postprandial network interactions for Reference Sleep + Exercise (RSX) and Short Sleep + Exercise (SSX). **(A)** Matrix elements in the correlation matrix represent pairwise coupling strength between each possible pair of HRV and LDL parameters (Pearson correlation coefficient; see Methods). Non-significant correlations are represented in green. Color code is shown in vertical color bars. **(B)** Nodes in the physiological network represent the different HRV and LDL parameters, and the network links correspond to the correlation matrix elements, reflecting the coupling strength between HRV and LDL parameters. Links strength is marked by line color and width and are divided into six types: strong positive links (Pearson coefficients >0.8), intermediate positive links (0.6 < Pearson coefficients < 0.8), weak positive links (0.4 < Pearson coefficients < 0.6); weak negative links (−0.4 > Pearson coefficients > −0.6); intermediate negative links (−0.6 > Pearson coefficients > −0.8), and strong negative links (Pearson coefficients < −0.8). HRV, heart rate variability; LDL, Low-density lipoprotein; HF-1, High-frequency power at time point -1; SDNN-1, standard deviation of RR interval at time point-1; RMSSD-1, the root mean square of successive normal RR interval differences at time point-1; TP-1, total power at time point-1; LF-1, low-frequency at time point-1.

**FIGURE 3:**
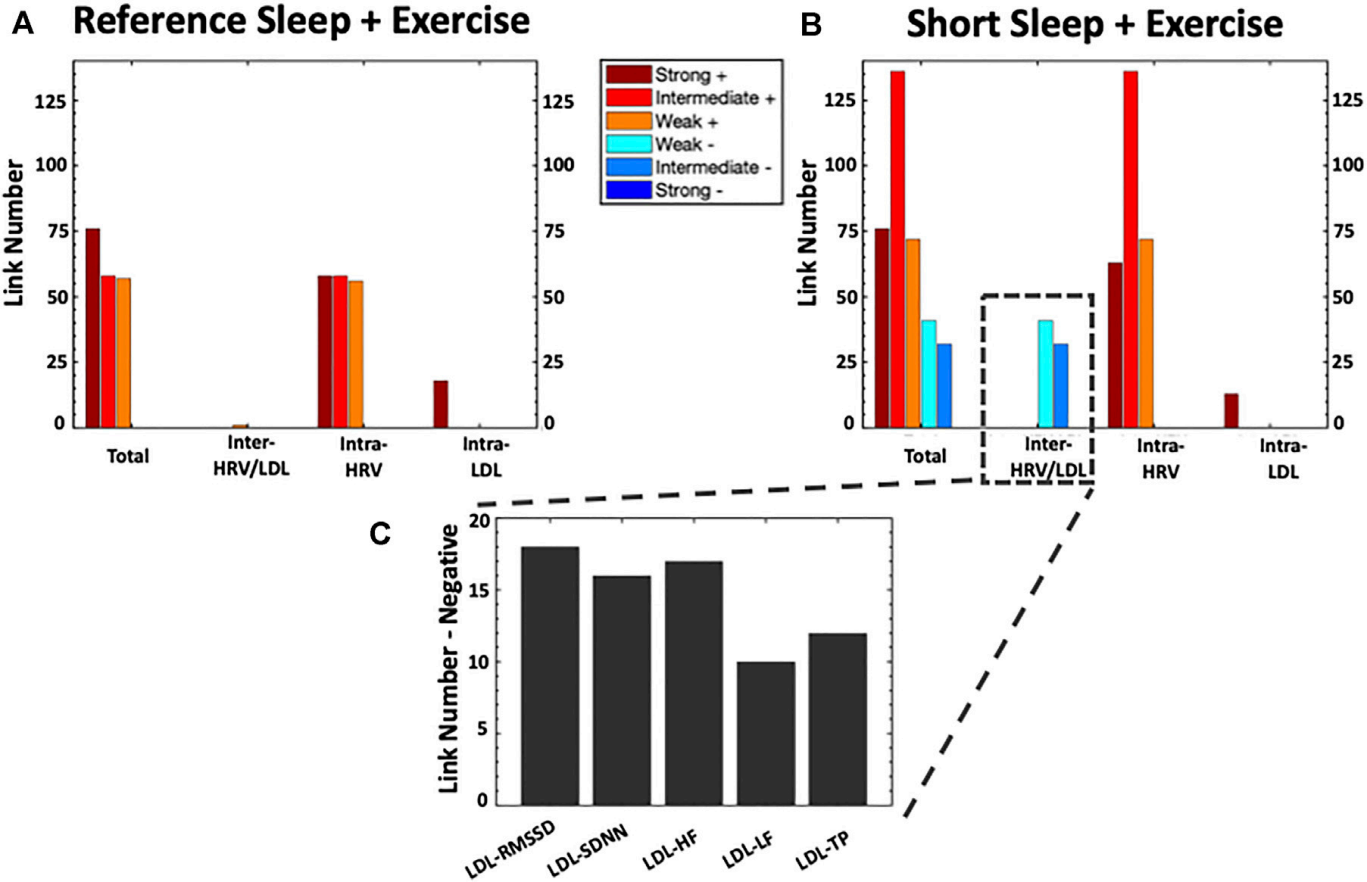
Bar Charts Panel representing the number of links (i.e., significant correlations) within each physiological network for Reference Sleep + Exercise (RSX) and Short Sleep + Exercise (SSX). The heigh of the bars in **(A)** and **(B)** indicate 1) the total number of links in the entire physiological network, 2) the number of links between the HRV and LDL sub-networks (inter-HRV/LDL); (iii) the number of links within the HRV sub-network (intra-HRV); and (iv) the number of links within the LDL sub-network (intra-LDL). Panel **(C)** shows the details for the number of inter-HRV/LDL negative links. HRV, heart rate variability; LDL, Low-density lipoprotein; HF, High-frequency power; SDNN, standard deviation of RR interval; RMSSD, the root mean square of successive normal RR interval differences; TP, total power; LF: low-frequency.

To obtain the correlation analysis (Figure 2A), the Pearson correlation coefficient was used to calculate the correlations between all possible pairs of the aforementioned HRV and LDL parameters, including inter-HRV/LDL (between HRV and LDL), intra-HRV (within HRV) and intra-LDL (within LD) parameters. To visualize the information provided by the correlation analysis, we next mapped the previously obtained correlation analysis into one physiological network (Figure 2B). This graphical approach is essential to identify patterns in the postprandial network structure and to track the differences in network characteristics for the different experimental conditions. The physiological network was constructed utilizing only the statistically significant correlations in the correlation analysis. The physiological network was comprised by two sub-networks: the HRV and the LDL sub-networks, where color nodes (30 for HRV and seven for LDL) represent the different HRV and LPL parameters, and the network links correspond to the correlation analysis elements reflecting the coupling strength between a given pair of parameters. Links strength is marked by line color and width and are divided into six types: strong positive links (Pearson coefficients >0.8), intermediate positive links (0.6 < Pearson coefficients <0.8), weak positive links (0.4 < Pearson coefficients <0.6); weak negative links (−0.4 > Pearson coefficients > −0.6); intermediate negative links (−0.6 > Pearson coefficients > −0.8), and strong negative links (Pearson coefficients < −0.8). With the aim of quantifying the interactions within the physiological network, we computed the number of links (i.e., number of significant correlations; Figure 3). Specifically, we calculated 1) the total number of links in the entire physiological network, 2) the number of links between the HRV and LDL sub-networks (inter-HRV/LDL); 3) the number of links within the HRV sub-network (intra-HRV); and 4) the number of links within the LDL sub-network (intra-LDL). Correlation matrices and physiological networks were processed and obtained by means of Matlab R2016b (Mathworks, Natik, MA, United States). The visualization framework used in our results is based on previous studies analyzing network interactions among physiological systems during different physiological states (Bashan et al., 2012; Bartsch et al., 2015; Lin et al., 2020; Prats-Puig et al., 2020) ***.***

## 3 Results

From the 30 individuals who signed consent participation forms, only 15 participants were able to adhere to study’s requirements and/or completed the study. Baseline screening of anthropometric and physiological characteristics are presented on Table 1. No differences at *p* = 0.05 were observed between conditions for the pre-experimental collected data of sleep, diet, and physical activity.

**TABLE 1:** Baseline screening.

Variable	Mean ± SE	Min	Max
Age (yrs.)	31 ± 5	24	40
Height (cm)	179.3 ± 6.6	167.6	187.9
Weight (kg)	83.3 ± 10.9	70.7	105.7
BMI (kg/m^2^)	25.8 ± 2.7	21.1	29.9
%BF	21.0 ± 6.2	11.4	35.3
Max VO_2_ (L/min)	4.0 ± 0.7	3.2	5.6
Max VO_2_ (ml/kg/min)	49.1 ± 8.2	35.5	65.6
Resting HR (bpm)	55 ± 7	42	63
Resting MAP (mmHg)	85 ± 10	70	100
PSQI	4 ± 0.9	2	5

All values are presented as mean ± standard error. VO_2_, volume of oxygen consumption; HR, heart rate; %BF, percent body fat; BMI, body mass index; MAP, mean arterial pressure was calculated as systolic blood pressure plus 2/3 of diastolic blood pressure; PSQI, Pittsburgh sleep quality index.

Experimental data for sleep and physical activity are presented in Table 2.

**TABLE 2:** Sleep and physical activity levels—conditions.

Variable	RSX	SSX
SL	8:11:04 ± 1:01:48	3:18:09 ± 0:52:02*
SD	6:57:09 ± 0:47:38	2:39:30 ± 0:40:11*
SLE	86 ± 8	81 ± 12
EE	2.8 ± 2.2	2.5 ± 2.0
Sedentary	0.8 ± 0.1	0.7 ± 0.1
Light	0.2 ± 0.1	0.2 ± 0.1
Moderate	0.1 ± 0.0	0.1 ± 0.0

All values and presented as mean ± standard error. Means with * are significantly different. Values are significant at *p* < 0.05. Sleep time values are in h:mm:ss format, except sleep efficiency that is in percentage. SL, sleep plus laying down in hours and minutes; SD, sleep duration in hours and minutes; SLE, sleep efficiency expressed as percentage of sleep duration over laying down time; Activity levels are labeled as Sedentary (up to 1.5 METs), Light (1.5–3.0 METs), and Moderate (3.0–6.0 METs); METs, metabolic equivalents; EE, energy expenditure in METs; RSX, reference sleep and high-intensity interval exercise condition; SSX, short and disrupted sleep and high-intensity interval exercise condition—acute partial sleep deprivation.

Experimental data for exercise are presented in Table 3. Percentage of coefficients of variation (CV%) for LDL cholesterol were calculated for RSX and SSX for all seven timepoints. The CV% for RSX was 9.8% and for the SSX was 10.5%, respectively.

**TABLE 3:** Exercise data.

Trials	Baseline	RSX	SSX
Weight (kg)	83.3 ± 10.9	83 ± 11.5	83 ± 11.5
Max Exercise VO_2_ (L/min)	-	4 ± 0.6	4 ± 0.6
Max Exercise VO_2_ (ml/kg/min)	-	46 ± 8.1	45 ± 8.1
Avg Exercise VO_2_ (L/min)	-	3 ± 0.4	3 ± 0.5
Avg Exercise VO_2_ (ml/kg/min)	-	33 ± 5.1	33 ± 5.8
Resting HR (bpm)	55 ± 7.0	58 ± 13.0	58 ± 10.0
Avg Exercise HR (bpm)	-	153 ± 12.0	150 ± 12.0
90% VO_2_ reserve (ml/kg/min)	-	42 ± 7.3	41 ± 7.3
40% VO_2_ reserve (ml/kg/min)	-	20 ± 3.2	20 ± 3.2
Resting MAP (mmHg)	85 ± 10.0	85 ± 8.0	83 ± 7.0
Exercise Duration (min)	-	24 ± 2.6	24 ± 2.7

All values are presented as mean ± standard error. Means with * are significantly different. Values are significant at *p* < 0.05. VO_2_, volume of oxygen consumption; HR, heart rate; MAP, mean arterial pressure was calculated as systolic blood pressure plus 2/3 of diastolic blood pressure; RSX, reference sleep and high-intensity interval exercise condition; SSX, short and disrupted sleep and high-intensity interval exercise condition—acute partial sleep deprivation.

Figure 2 shows the correlation matrices and physiological networks representing postprandial network interactions for RSX and SSX conditions. Matrix elements in the correlation analysis represent pairwise coupling strength between each possible pair of HRV and LDL parameters. Nodes in the physiological network represent the different HRV and LDL parameters, and the network links correspond to the correlation analysis elements, reflecting the coupling strength between HRV and LDL parameters. We observed a clearly differentiated network of postprandial interactions between RSX and SSX due to the irruption of negative links in the SSX network.

As depicted in Figure 3, the total number of links in the physiological network increased by 86.9% under SSX compared to RSX (356 vs 191 links) due to 1) the manifestation of weak and intermediate negative inter-HRV/LDL links, and 2) an increase of positive intra-HRV links.

No inter-HRV/LDL links were observed between the HRV and LDL sub-networks in RSX. However, a remarkable incursion of weak and intermediate negative links was observed between the HRV and LDL sub-networks in SSX. These negative association were present for all HRV parameters, with higher number of links between LDL and RMSSD, SDNN and HF (see Figure 2C).

Regarding the intra-HRV and intra-LDL links, the HRV sub-network was characterized by an increased number of intermediate positive links in SSX (136%) compared to RSX. No remarkable differences were observed between RSX and SSX for intra-LDL links in the LDL sub-network.

## 4 Discussion

This study investigated postprandial network interactions between autonomic regulation through HRV, and lipemia through low-density lipoprotein (LDL) cholesterol in response to APSD and after HIIE. We reported an irruption of inter-HRV/LDL negative links in the physiological network of SSX compared to RSX, which a priori we defined as the healthy network. The presence of weak and intermediate negative links between the HRV and LDL sub-networks in SSX reflected the impact of sleep deprivation on the autonomic regulation and lipemia. Further, increased connectivity was noted within the HRV sub-network in SSX, with no differences documented for the LDL sub-network. These findings revealed the inability of HIIE to remain cardioprotective under APSD state, and underlie the need to further investigate the effects of APSD and HIIE on the interactions among physiological systems.

The presence of negative links between HRV and LDL for SSX is supported by evidence indicating that impaired balance of the ANS may be the mechanistic explanation linking APSD to CVD (Zhong et al., 2005; Tobaldini et al., 2014). Literature indicates that APSD increases the sympathoadrenal influence, has a greater impact on metabolic and cardiovascular functions that result to increased catecholamine levels, dampened glucose metabolism, increased heart rate (HR) and blood pressure (BP) (Gottlieb et al., 2006; Hall et al., 2008; Meerlo et al., 2008). In addition, it alters the hypothalamic-pituitary-adrenal (HPA) axis activity towards to a higher glucocorticoid release and subsequent systemic inflammatory and oxidative stress response (Meerlo et al., 2008). Therefore, it is apparent that physiologically speaking, APSD has detrimental health effects, which may be observed after very short-term exposure (Alvarez and Ayas, 2004; Gangwisch et al., 2006; Atkinson and Davenne, 2007; Knutson et al., 2007; Buxton and Marcelli, 2010; Centers for Disease and Prevention, 2011; Dettoni et al., 2012; Wu et al., 2012; Calvin et al., 2014). Moreover, acute exercise induces changes in autonomic tone and disturbs the ANS (Thompson et al., 2007; Fukuda et al., 2015), with the HIIE to disturb even more the ANS, and possible to contributing to an unhealthy cardiometabolic status, even after a single episode of HIIE (Besnier et al., 2017).

Considering RSX as the reference healthy and functional network, the HRV sub-network for SSX was characterized by an increment of intermediate positive links, reflecting an increased connectivity due to a reduced sleep time. As previously described (Balague et al., 2020) both underexpressed (weak) and overexpressed network connectivity could reflect unfunctional/pathological states. More specifically, overexpressed/excessive connectivity as observed within the HRV sub-network for SSX, could be associated with a transitory underexpression of coupling network connectivity (i.e., imbalance: some processes are overexpressed and others underexpressed). An example of such imbalance is the rigidity and reduction of diversity potential provoked by exercise-induced fatigue (Vazquez et al., 2016; Vazquez et al., 2020). Similarly, some pathological conditions (e.g., neuro-muscular disorders) could increase the density and/or strength of interactions among certain nodes, pushing the system toward a rigid order which, in turn, could reduce its adaptability to environmental constraints (Ivanov et al., 1998; Ivanov et al., 2001; Stergiou et al., 2006; Stergiou and Decker, 2011). The results of this study support the notion of characterizing a healthy network based on the number of network links.

Several sleep protocols have examined the sleep deprivation effects on healthy individuals in respect to cardiovascular changes and autonomic control via HRV (Tobaldini et al., 2014; Tobaldini et al., 2017). Some reported no differences in HRV with just 4 h sleep (Muenter et al., 2000), while other showed an increase in sympathetic activity (SA) as demonstrated in total decreased HRV, increased low-frequency (LF), and decreased high-frequency (HF) compared to control of 8 h sleep (Dettoni et al., 2012). Our results are in agreement with those that showed an impact of short sleep duration on autonomic regulation (Dettoni et al., 2012) as we reported negative associations for all the HRV parameters and lipemia.

It is important though to mention that studies that examine sleep, HRV, exercise and cardiometabolic health due to differences in the employed research designs, settings, examined variables, sleep durations, sample characteristics etc., yield heterogenous results that are difficult to compared and provide clear and comprehensive outcomes (Malik, 1996; Elsenbruch et al., 1999; Kaikkonen et al., 2008; Al Haddad et al., 2009; Dettoni et al., 2012; Myllymaki et al., 2012; Uchida et al., 2012; Heydari et al., 2013; Tobaldini et al., 2013; Oda and Shirakawa, 2014; Michael et al., 2017; Tobaldini et al., 2017; van Leeuwen et al., 2018; Abreu et al., 2019; Barroso et al., 2019; Costa et al., 2019; Schneider et al., 2019; Vitale et al., 2019). Therefore, research questions related to the immediate HRV responses after acute HIIE following reference sleep and APSD are still under investigation. Previous work from our lab has examined such research questions (Papadakis et al., 2020; Papadakis et al., 2021a; Papadakis et al., 2021b) using the traditional reductionistic approach focusing on a single physiological system and investigated the mechanistic interactions with other single systems by reducing complex multicomponent systems on their respective parts (Machamer et al., 2000; Bechtel and Richardson, 2010; Balague et al., 2020). We reported that HIIE was cardioprotective and APSD did not influence the HRV (Papadakis et al., 2021a), neither the postprandial endothelial function (Papadakis et al., 2020), nor the exercise performance (Papadakis et al., 2021b).

Investigating though the same research questions under the Network Physiology of Exercise perspective we showed that APSD and HIIE had an impact on the HRV and LDL. It seems though that our previous investigations were not able to capture the synchronization and integration among autonomic nervous and cardiometabolic systems. Recent work from Ivanov and Bartsch, (2014) and Ivanov et al., (2017) highlighted the fact that physiological states emerge due to specific network organization, topology, and their respective network of dynamic interactions. Moreover, such network of dynamic interactions is moving past the concepts of interconnectivity across of physiological systems and the statistical inference of static associations that govern physiological states (Sieck, 2017; Head, 2020). As such, our previous analyses (Papadakis et al., 2020; Papadakis et al., 2021a; Papadakis et al., 2021b), failed to provide a comprehensive understanding of the dynamic interaction of the involved physiological systems and their subsystems to generate dynamic integrated response at the organism level (Balague et al., 2020). The findings of this study reinforce previous works suggesting that the commonly utilized physiological parameters (e.g., VO_2_max) provide little information on the nature of the dynamic interactions among physiological systems and their common role in an integrated network. Coordinative variables, such as cardio-respiratory coordination or other psychophysiological parameters, can detect qualitative changes related to the coordinated activity among physiological systems, and their changes under exercise-related constraints (Balagué et al., 2013; Esquius et al., 2019; Garcia-Retortillo et al., 2019).

A major limitation of this study is that it cannot be considered as a true network analysis, as no time series of physiological variables were recorded and analyzed. Note that to capture interactions among physiological systems, time series analysis and the detection of coordinative variables would be the most appropriate strategy. This study was not initially conceived to investigate postprandial network interactions between autonomic regulation and lipemia, but to mimic real life settings between APSD and HIIE under the traditional framework of Exercise Physiology. The applied correlation analysis does not have the power to identify dynamic interactions between the investigated physiological systems. Therefore, this study has inherited all the limitations of the traditional Exercise Physiology framework, that is, the tacit assumption that results obtained by a sample can be generalized to a population level based on the representative observed changes of a “typical” (i.e., average) individual. This assumption though can be true only if the system is ergodic and its evolution in time is stationary and the structure of the interindividual multivariate dynamics is the same across all individuals (Balague et al., 2020). Moreover, since this is reanalysis of data collected for another purpose, it carries the limitations of our previous investigations (e.g., absence of APSD and no HIIE, only apparently healthy men who were good sleepers, environmental stress and factors outside of controlled laboratory settings, indirect method of measuring the cardiac autonomic activity, time of day and chronotype of our sample) (Papadakis et al., 2020; Papadakis et al., 2021a; Papadakis et al., 2021b). At the same time though, this study’s strength is the application of the NPE approach to examine a research question with controversial results when examined through the traditional Exercise Physiology framework. This study is providing preliminary evidence on the sensitivity of the NPE approach to capture interactions among different physiological systems. In this line, further research utilizing time series of physiological variables (Garcia-Retortillo et al., 2020) is needed to investigate the effects of APSD and HIIE on postprandial network interactions.

## 5 Conclusion

The human organism is composed of various integrated networks and sub-networks of interconnected organs, systems, and functions, a disruption or failure of one system can trigger a cascade of failures that can be manifested as a disease state (Ivanov and Bartsch, 2014; Goldman et al., 2015; Liu et al., 2015; Ivanov et al., 2017; Sieck, 2017; Liu and Chen, 2019; Balague et al., 2020; Barajas-Martinez et al., 2020; Corkey and Deeney, 2020). We investigated postprandial network interactions between autonomic regulation through HRV, and lipemia through LDL cholesterol in response to APSD and HIIE. We observed an increase of inter-HRV/LDL negative links in the SSX physiological network compared to RSX. These results reflected the impact of sleep deprivation on the autonomic regulation and lipemia and, revealed the inability of HIIE to remain cardioprotective under APSD.

### 5.1 Resource Identification Initiative

To take part in the Resource Identification Initiative, please use the corresponding catalog number and RRID in your current manuscript. For more information about the project and for steps on how to search for an RRID, please click here.

## Data Availability

The data analyzed in this study is subject to the following licenses/restrictions No restrictions applied to the dataset. Requests to access these datasets should be directed to zpapadakis@barry.edu.
